# Clinical description and outcome of overall varicella-zoster virus-related organ dysfunctions admitted in intensive care units: the VAZOREA cohort study

**DOI:** 10.1186/s13613-024-01270-w

**Published:** 2024-03-29

**Authors:** Jolan Malherbe, Pierre Godard, Jean-Claude Lacherade, Valentin Coirier, Laurent Argaud, Hervé Hyvernat, Francis Schneider, Julien Charpentier, Florent Wallet, Juliette Pocquet, Gaëtan Plantefeve, Jean-Pierre Quenot, Pierre Bay, Agathe Delbove, Hugues Georges, Tomas Urbina, David Schnell, Charlène Le Moal, Matthieu Stanowski, Corentin Muris, Maud Jonas, Bertrand Sauneuf, Olivier Lesieur, Amaury Lhermitte, Laure Calvet, Ines Gueguen, Damien du Cheyron

**Affiliations:** 1 Normandie Univ, UNICAEN, CHU de Caen Normandie, Médecine Intensive – Réanimation, Caen, 14000 France; 2 Service de Médecine Intensive – Réanimation, CHU Bordeaux site Pellegrin, Bordeaux, France; 3 Médecine Intensive – Réanimation, CH La Roche Sur Yon, La Roche Sur Yon, France; 4 Service de Médecine Intensive – Réanimation, CHU de Rennes, Rennes, 35000 France; 5 Service de Médecine Intensive – Réanimation, Hôpital Edouard Herriot, Hospices civils de Lyon, Université de Lyon, Université Claude Bernard Lyon 1, Faculté de Médecine Lyon-Est, Lyon, France; 6 Service de Médecine Intensive – Réanimation, Université Côte d’Azur (UCA), CHU de Nice, 151 route Saint Antoine de Ginestière, Nice, 06200 France; 7 Médecine Intensive – Réanimation, Hôpital de Hautepierre, Hôpitaux Universitaires de Strasbourg et Unistra, Strasbourg, France; 8 Service de Médecine Intensive – Réanimation, Centre-Université Paris Cité, Hôpital Cochin, Assistance Publique-Hôpitaux de Paris, Paris, 75014 France; 9 Médecine Intensive – Réanimation, CHU Lyon Sud, Pierre Benite, France; 10 RESHAPE Research on healthcare performance, U1290, Université Claude Bernard Lyon 1, Lyon, France; 11 Médecine Intensive – Réanimation, CHR Orléans, Orléans, France; 12 Service de Réanimation, CH Argenteuil, Argenteuil, France; 13 Department of Intensive Care, Burgundy University Hospital, Dijon, France; 14 Service de Médecine Intensive – Réanimation, AP-HP Assistance Publique Hôpitaux de Paris, Hôpitaux universitaires Henri Mondor, DMU Médecine, Créteil, 94010 France; 15 UPEC Université Paris-Est Créteil, INSERM, Unité U955, Equipe 18, Créteil, 94010 France; 16 Service de réanimation polyvalente, CHBA Vannes, Vannes, France; 17 Service de réanimation polyvalente, Centre hospitalier de Tourcoing, Tourcoing, 59200 France; 18 Service de Médecine Intensive – Réanimation, Hôpital Saint-Antoine, Assistance Publique- Hôpitaux de Paris, Paris, 75012 France; 19 Réanimation Polyvalente et USC, CH Angoulême, Angoulême Cedex 9, Angoulême, 19959 France; 20 Service Réanimation/USC, Centre Hospitalier du Mans, Le Mans, 72037 France; 21 Médecine Intensive – Réanimation, CHRU de Nancy, Nancy, France; 22 Université de Poitiers, CHU de Poitiers, Médecine intensive Réanimation, 2 rue de la miletrie, Poitiers, 86000 France; 23 Service Médecine Intensive – Réanimation/USC, Centre hospitalier de Saint-Nazaire, Saint-Nazaire, 44600 France; 24 Service de Réanimation polyvalente, Centre Hospitalier Public du Cotentin, Cherbourg en Cotentin, 50100 France; 25 Centre Hospitalier Saint-Louis, Réanimation polyvalente, La Rochelle, 17019 France; 26 Hôpital Universitaire Félix Guyon, Réanimation polyvalente, Allée des Topazes, Saint-Denis, La Réunion, 97400 France; 27 Service de Médecine Intensive et Réanimation, CHU de Clermont-Ferrand, Clermont- Ferrand, France; 28 Service de réanimation médicale, CHRU de Lille, Lille, France

**Keywords:** Varicella-Zoster virus, Intensive care units, Encephalitis, Pneumonia, Immunocompromised host

## Abstract

**Background:**

Due to aging population and increasing part of immunocompromised patients, a raise in life-threatening organ damage related to VZV can be expected. Two retrospective studies were already conducted on VZV in ICU but focused on specific organ injury. Patients with high-risk of VZV disease still must be identified. The objective of this study was to report the clinical features and outcome of all life-threatening VZV manifestations requiring intensive care unit (ICU) admission. This retrospective cohort study was conducted in 26 French ICUs and included all adult patients with any life-threatening VZV-related event requiring ICU admission or occurring in ICU between 2010 and 2019.

**Results:**

One-hundred nineteen patients were included with a median SOFA score of 6. One hundred eight patients (90.8%) were admitted in ICU for VZV disease, leaving 11 (9.2%) with VZV disease occurring in ICU. Sixty-one patients (51.3%) were immunocompromised. Encephalitis was the most prominent organ involvement (55.5%), followed by pneumonia (44.5%) and hepatitis (9.2%). Fifty-four patients (45.4%) received norepinephrine, 72 (60.5% of the total cohort) needed invasive mechanical ventilation, and 31 (26.3%) received renal-replacement therapy. In-hospital mortality was 36.1% and was significantly associated with three independent risk factors by multivariable logistic regression: immunosuppression, VZV disease occurring in ICU and alcohol abuse. Hierarchical clustering on principal components revealed five phenotypically distinct clusters of patients: VZV-related pneumonia, mild encephalitis, severe encephalitis in solid organ transplant recipients, encephalitis in other immunocompromised hosts and VZV disease occurring in ICU. In-hospital mortality was highly different across phenotypes, ranging from zero to 75% (*p* < 0.001).

**Conclusion:**

Overall, severe VZV manifestations are associated with high mortality in the ICU, which appears to be driven by immunosuppression status rather than any specific organ involvement. Deciphering the clinical phenotypes may help clinicians identify high-risk patients and assess prognosis.

**Supplementary Information:**

The online version contains supplementary material available at 10.1186/s13613-024-01270-w.

## Background

Varicella-zoster virus (VZV) is a ubiquitous herpesvirus known to cause infections in humans, mostly during childhood [1–3]. In the Western world, VZV seroprevalence is approximately 80 to 90% of the adult population [4, 5]. After primary infection, VZV remains latent in the sensitive dorsal-root or cranial nerve ganglia from where it is able to reactivate during immunosuppression or aging [2, 3, 6]. Recent progress in the diagnosis and management of patients with malignancies or autoimmune diseases has increased the proportion of immunocompromised patients [7–12]. Together with the aging population, this phenomenon has led to an increased incidence of Herpes zoster [13–16]. Consequently, a raise in life-threatening organ damage related to VZV may be expected.

Long considered a mild disease, VZV-associated disease is now increasingly identified as life-threatening, leading to organ dysfunctions and intensive care unit (ICU) admissions [17, 18]. Indeed, pneumonia represents the most frequent and severe VZV-related organ involvement accounting for up to 16.3% of chickenpox cases in the adulthood, with a hospital mortality of 24% for those requiring ICU admission [1, 3, 19–22]. Varicella-zoster virus also represents an increasing cause of encephalitis being now recognized as the second encephalitis agent and the first in immunocompromised patients, representing 4 to 14% of total cases [23, 24]. In a recent nationwide cohort study, VZV encephalitis was associated with a 11% 3-month mortality and an unfavourable outcome in 69% of cases [25].

Despite an increasing incidence and proven severity, especially in immunocompromised patients [26], literature data on life-threatening VZV-related events are scarce and focus on specific organ injuries [22, 25, 27]. High-risk patients and their clinical presentations must still be identified as they may benefit from prompt antiviral therapy or prophylactic strategies.

Therefore, we conducted a multicentre cohort study aiming to report the characteristics and prognosis of all patients with any severe VZV-related organ injury requiring ICU admission.

Partial results of this cohort study were reported at the French Intensive Care Society annual congress and published as abstract [28].

## Methods

The present study was approved by the French Intensive Care Society ethics committee (CE SRLF #20–38). Collection of patient data in a database and their analysis was authorized by the French data protection agency (#920460). In accordance with French law on retrospective anonymized data, a waiver for informed consent was obtained.

The study complies with the Strengthening the Reporting of Observational Studies in Epidemiology (STROBE) statement guidelines (Additional file 1).

### Study design

This retrospective multicentre cohort study was conducted in 26 French ICUs (Additional file 2: Table 1). Adult patients ($\ge$ 18 years of age) with severe VZV manifestations admitted from January 2010 to December 2019 were identified from the electronic hospital databases based on codes B01, B02, G02, G05 and J17.1, from the International Classification of Diseases – 10th revision. Severe VZV manifestations were defined as requiring ICU admission. Cutaneous herpes zoster was not included. VZV disease was defined as occurring in ICU when the first symptoms appeared more than 48 h after ICU admission and led to new organ dysfunction. All cases were reviewed by local investigators and classified based on medical charts.

### Data collection and definitions

Patient data were extracted from the medical records of participating centres. Baseline characteristics including demographics, chronic comorbidities, immunocompromised status, use of antiviral prophylaxis, other infections, main reason for ICU admission, sequential organ failure assessment (SOFA) and simplified acute physiology (SAPS II) scores [29, 30], VZV-related organ injury, method of diagnosis, and routine laboratory parameters were collected on admission. Alcohol abuse was defined according to French guidelines on alcohol consumption (at-risk use of alcohol) [31]. Immunosuppression was defined as ongoing solid tumour or cured less than five years before admission, any haematologic malignancy, autoimmune disease, solid organ transplant, primary immune deficit, HIV infection, use of systemic corticosteroids ($\ge$5 mg/day of prednisone or equivalent) or immunosuppressive drugs. VZV-related pneumonia was considered in case of acute respiratory failure and no other cause deemed as probable as VZV. Acute respiratory distress syndrome (ARDS) was defined according to the Berlin definition [32]. Encephalitis was considered in patients with signs of central nervous system involvement (consciousness disorder – which is the major criterion for encephalitis based on International Encephalitis Consortium guidelines [33] –, seizures or focal signs). Sepsis and septic shock were defined according to the Sepsis-3 definition [34]. Hepatitis was defined as an elevation of alanine and aspartate aminotransferases as defined by the 2017 guidelines of the American College of Gastroenterology [35]. VZV-associated pancreatitis was defined according to the 2012 revised Atlanta definition for acute pancreatitis [36]. During ICU stay, collection of data included in-ICU management, ICU-acquired infections, and outcomes (ICU and hospital mortality, and occurrence of acute kidney injury as defined by the Kidney Disease Improving Global Outcome criteria based on creatinine [37]).

### Statistical analysis

Continuous variables were expressed as median (interquartile range, IQR), and categorical variables as numbers (percentages). Between-group comparisons were performed by Mann-Whitney or Kruskal-Wallis tests for continuous variables, and Fisher exact test for categorical variables.

Two multidimensional unsupervised factorial methods were used for description of data and clustering of patients: first multiple component analysis (MCA), used as pre-processing, then hierarchical clustering on principal components (HCPC). Factorial analyses are descriptive statistical methods representing data as multidimensional scatter plots that are used to describe correlations between variables or individuals. The variables used for these analyses were age and gender, SOFA score, comorbidities, immunosuppression status (as previously defined), VZV disease occurring in or outside ICU, organ injury, use of life-sustaining therapies, occurrence of ICU-acquired infection, ICU and hospital mortality, lymphocyte and platelet counts on admission.

As HCPC can only be used with quantitative data, MCA was used to pre-process qualitative variables. Data processed using MCA were then subjected to HCPC, using Ward’s method to merge patients into clusters. The number of clusters was defined to minimize loss of inertia. A graphical representation of patients and clusters was produced by projecting the patients on a factorial plan, using the first two dimensions to summarize the maximum of data variability. As a diagnosis relying only on clinical examination could be a source of bias, a sensitivity factorial analysis was built after exclusion of these patients.

To identify risk factors associated with hospital mortality, a multivariable logistic regression was performed, with a ratio of 1 variable per 8 events. Three variables were selected based on the existing literature (age, SOFA score and immunocompromised status) [22, 25, 27]. After univariable logistic regression, the two variables most associated with hospital mortality were also included in a multivariable model.

All analyses were performed with R version 4.0.5 (R foundation for Statistical Computing, Vienna, Austria; https://www.R-project.org/) with FactoMineR and Factoshiny packages for MCA and HCPC. All tests were two-sided, and a *p* value < 0.05 was considered statistically significant.

## Results

### Study population

From January 2010 to December 2019, 222,118 patients were admitted in the 26 participating ICUs and 119 were included in the study (0.054%) with a median age of 66 years (Additional file 2: Fig. 1). Main characteristics of cases are presented in Table 1.

Sixty-one patients (51.3%) were immunosuppressed. The main causes of immunosuppression were haematologic malignancies with a majority of non-Hodgkin lymphoma (Additional file 2: Table 2), autoimmune diseases and solid organ transplant. Among patients treated with immunosuppressive drugs, 33.3% (8 out of 24) were receiving more than one therapy (Additional file 2: Table 2).

**Table 1:** Main features of the 119 patients based on ICU admission with or without VZV disease

Characteristics	Overall (*n* = 119)	Admission with VZV disease (*n* = 108)	VZV disease occurring in ICU (*n* = 11)	*P*
Age (years), *median [IQR]*	66 [45–75]	45.5 [43.75–75.25]	66 [46.5–74]	*0.96*
Female sex at birth, *n (%)*	46 (38.7)	44 (40.7)	2 (18.2)	*0.2*
SOFA score	6 [2–9]	5.5 [2–9]	8 [4–10.5]	*0.47*
SAPS II score	40 [26–60.25]	40 [26–61]	40 [29.5–52]	*0.87*
Hypertension, *n (%)*	51 (42.9)	45 (41.7)	6 (54.5)	*0.53*
Liver cirrhosis, *n (%)*	10 (8.4)	8 (7.4)	2 (18.2)	*0.23*
Alcohol abuse, *n (%)*	18 (15.1)	14 (13)	4 (36.4)	*0.06*
Chronic kidney disease, *n (%)*	19 (16)	16 (14.8)	3 (27.3)	*0.38*
Type 2 diabetes mellitus, *n (%)*	18 (15.1)	16 (14.8)	2 (18.2)	*0.67*
Immunocompromised^1^, *n (%)*	61 (51.3)	54 (50)	7 (63.6)	*0.53*
Solid tumour, *n (%)*	12 (10.1)	9 (8.3)	3 (27.3)	*0.08*
Haematologic malignancy, *n (%)*	23 (19.3)	23 (21.3)	0 (0)	*0.12*
Autoimmune disease, *n (%)*	18 (15.1)	17 (15.7)	1 (9.1)	*1*
Solid-organ transplant, *n (%)*	13 (10.9)	10 (9.3)	3 (27.3)	*0.1*
Corticosteroids, *n (%)*	27 (22.7)	24 (22.2)	3 (27.3)	*0.71*
Immunosuppressive drugs, *n (%)*	24 (20.2)	21 (19.4)	3 (27.3)	*0.69*
HIV, *n (%)*	4 (3.4)	3 (2.8)	1 (9.1)	*0.33*
Pregnancy, *n (%)*	4 (3.4)	3 (2.8)	1 (9.1)	*0.33*
Time between hospital presentation and ICU admission (days)	2 [0–5]	2 [0–5]	2 [0.5–20.5]	*0.34*
Time between first symptoms and ICU admission (days)	-4 [-7 – -2]	-4 [-7 – -2]	32.5 [22–54]	*< 0.001*
***Main reason for ICU admission, n (%)***				*0.13*
Neurologic	51 (42.9)	49 (45.4)	2 (18.2)	
Respiratory	45 (37.8)	39 (36.1)	6 (54.5)	
Circulatory	4 (3.4)	3 (2.8)	1 (9.1)	
Multiple organ failure	10 (8.4)	10 (9.3)	0 (0)	
Other	9 (7.6)	7 (6.5)	2 (18.2)	
***VZV-related organ injury, n (%)***				
VZV vesicular skin rash	89 (74.8)	81 (75)	8 (72.7)	*1*
Encephalitis	66 (55.5)	62 (57.4)	4 (36.4)	*0.21*
Pancreatitis	5 (4.2)	5 (4.6)	0 (0)	*1*
Hepatitis	11 (9.2)	9 (8.3)	2 (18.2)	*0.27*
Pneumonia	53 (44.5)	48 (44.4)	5 (45.5)	*1*
ARDS	26 (21.8)	23 (21.3)	3 (27.3)	*0.7*
***Laboratory parameters on ICU admission***				
Lymphocyte count (/mm^3^)	845 [378.25–1670]	865 [372.75–1730]	700 [500–935]	*0.39*
Platelets (G/L)	156.5 [90–240.25]	147 [91–231]	261 [162–283]	*0.08*
CSF leucocyte count (/mm^3^)^2^	76 [15.5–223]	84.5 [16–262.25]	13 [7.5–44.5]	*0.16*
***In-ICU management***				
Norepinephrine, *n (%)*	54 (45.4)	47 (43.5)	7 (63.6)	*0.22*
Invasive mechanical ventilation, *n (%)*	72 (60.5)	63 (58.3)	9 (81.8)	*0.11*
Duration of invasive mechanical ventilation, *n (%)*	10 [4.75–20]	9 [4–19]	23 [17–51]	*0.003*
Renal replacement therapy, *n (%)*	31 (26.3)	25 (23.4)	6 (54.5)	*0.04*
Antiviral therapy, *n (%)*	117 (98.3)	107 (99.1)	10 (90.9)	*0.09*
Other ICU-acquired infection, *n (%)*	44 (37)	37 (34.6)	7 (63.6)	*0.1*
Withholding or withdrawing of life sustaining therapy, *n (%)*	15 (12.6)	13 (12)	2 (18.2)	*0.68*
***Prognosis***				
ICU mortality, *n (%)*	37 (31.1)	30 (27.8)	7 (63.6)	*0.03*
Hospital mortality, *n (%)*	43 (36.1)	35 (32.4)	8 (72.7)	*0.02*

^1^Defined as ongoing solid tumour or cured less than 5 years prior, hematologic malignancy, autoimmune disease, solid organ transplant, primary immune deficit, HIV infection, corticosteroids, or immunosuppressive drugs

^2^Four missing values

VZV-related organ injury was dominated by encephalitis (66 cases representing 55.5% of the cohort) and pneumonia (53 patients, 44.5%), including 26 patients (21.8%) presenting with ARDS. Pancreatitis did not appear solely but rather in a context of multiple organ injury. Considering hepatitis, only three patients presented with isolated liver injury, the remaining eight took part of a multiple organ injury. Six out of eleven patients developed severe hepatitis, defined by a prothrombin time below 50%. A typical vesicular skin rash was present in 74.8% of cases. Twenty-three patients (19.3%) were included in the study based on clinical examination only (without virological detection of VZV).

Eleven patients were classified as VZV disease occurring in ICU. Among them, two demonstrated cytopathic effect on biopsy. There was no difference with patients admitted in ICU with VZV disease, except for a prolonged stay in ICU before the onset of VZV disease (32.5 days [22–54]), a prolonged duration of mechanical ventilation (9 [4–19] vs. 23 [17–51] days, *p* = 0.003) and a higher rate of renal replacement therapy (RRT) (25 (23.5%) vs. 6 (54.5%), *p* = 0.04) (Table 1).

### In-ICU management

On ICU admission, patients presented with severe illness as indicated by high SAPS II and SOFA scores (40 [26–60.25] and 6 [2–9] respectively, Table 1).

The median time from hospital presentation to ICU admission was 2 days (IQR 0–5). A longer time delay was significantly associated with hospital mortality by univariable analysis (1 [0–3] vs. 2 [1–11.5] days, *p* = 0.002). Among the 117 patients treated with antiviral therapy, 113 received acyclovir, 3 received valacyclovir and only one patient received ganciclovir (concomitant CMV viraemia) (Additional file 2: Table 2). By univariable analysis, survivors received their first antiviral infusion sooner (1 [0–3] vs. 2 [1–6.75] days, *p* = 0.01) (Table 2).

**Table 2:** Univariable and multivariable analyses of factors associated with in-hospital mortality

Univariable analysis				Multivariable analysis	
Characteristics	Survivors (*n* = 76)	Nonsurvivors (*n* = 43)	*P*	OR [95% CI]	*P*
Age, *median [IQR]*	63.5 [41.5–74]	70 [51.5–76.5]	*0.05*	1.02 [0.99–1.04]	*0.23*
SOFA score	4.5 [2–8]	8 [4–11]	*0.01*	1.09 [0.98–1.21]	*0.12*
SAPS II score	34 [21.5–53]	53.5 [36.75–68.25]	*< 0.001*		
Hypertension, *n (%)*	27 (35.5)	24 (55.8)	*0.04*		
Alcohol abuse, *n (%)*	6 (7.9)	12 (27.9)	*0.006*	4.48 [1.36–16.27]	*0.02*
Immunocompromised^1^, *n (%)*	30 (39.5)	31 (72.1)	*0.001*	3.43 [1.4–8.99]	*0.01*
Type 2 diabetes mellitus, *n (%)*	7 (9.2)	11 (25.6)	*0.03*		
Time between hospital presentation and ICU admission^2^ (days)	1 [0–3]	3 [1–11.5]	*0.002*		
VZV disease on ICU admission, *n (%)*	73 (96.1)	35 (81.4)	*0.02*		
VZV disease occurring in ICU, *n (%)*	3 (3.9)	8 (18.6)	*0.02*	4.5 [1.02–25.36]	*0.05*
***VZV-related organ injury, n (%)***					
Vesicular skin rash	61 (80.3)	28 (65.1)	*0.11*		
Encephalitis	41 (53.9)	25 (58.1)	*0.7*		
Pancreatitis	3 (3.9)	2 (4.7)	*1*		
Hepatitis	4 (5.3)	7 (16.3)	*0.1*		
Pneumonia	35 (46.1)	18 (41.9)	*0.7*		
ARDS	14 (18.4)	12 (27.9)	*0.25*		
***Laboratory values on ICU admission***					
Lactate (mmol/L)	1.2 [0.8–2.35]	1.7 [1.25–2.65]	*0.08*		
Leucocyte count (/mm^3^)	8800 [6225–11,850]	11,310 [7575–13,895]	*0.02*		
Lymphocyte count (/mm^3^)	895 [502.5–1650]	585 [285–1575]	*0.15*		
Platelets (G/L)	162 [96–239.5]	147 [78.5–238.5]	*0.85*		
CSF leucocyte count (/mm^3^)^3^	130 [30–330]	19 [6.75–74.75]	*0.004*		
CSF protein count (g/L)^3^	0.94 [0.61–1.8]	0.96 [0.65–2.32]	*0.49*		
***In ICU management***					
Norepinephrine, *n (%)*	27 (35.5)	27 (62.8)	*0.01*		
Dobutamine, *n (%)*	1 (1.3)	2 (4.7)	*0.3*		
Invasive mechanical ventilation, *n (%)*	37 (48.7)	35 (81.4)	*0.01*		
Renal replacement therapy, *n (%)*	12 (15.8)	19 (45.2)	*0.001*		
Antiviral therapy, *n (%)*	76 (100)	41 (97.6)	*0.36*		
Time between hospital presentation and first antiviral infusion^2^ (days)	1 [0–3]	2 [1–6.75]	*0.01*		
Systemic corticosteroids for VZV infection, *n (%)*	5 (6.8)	5 (12.2)	*0.52*		
ICU-acquired infection, *n (%)*	25 (32.9)	19 (45.2)	*0.23*		
>1 episode, *n (%)*	4 (5.3)	8 (19)	*0.03*	*2.57 [0.64–11.58]*	*0.19*

^1^Defined as ongoing solid tumour or cured less than 5 years prior, hematologic malignancy, autoimmune disease, solid organ transplant, primary immune deficit, HIV infection, corticosteroids, or immunosuppressive drugs

^2^VZV disease occurring in ICU excluded

^3^Four missing values

Fifty-four patients (45.4%) received norepinephrine, with a median maximum dosing of 0.42 µg/kg/min (IQR 0.22–0.95) and a median duration of 3 days (IQR 1–5). Respiratory support was used in 99 patients (83.9%), 72.7% of them (72 patients) needed invasive mechanical ventilation for a median duration of ten days (IQR 4.75–20). Most patients were intubated during their first day following ICU admission. RRT was used in 31 patients (26.3%) (Table 1).

Acyclovir median dosing regimen of 30 mg/kg/day (IQR 30–45). The regimen was in compliance with current French guidelines [38] in 68.5% of cases (Additional file 2: Table 2). Ten patients (8.7%) received systemic corticosteroids as a rescue therapy for VZV-related events, which was not associated with hospital prognosis (Table 2).

### Prognosis

Overall, 37 patients died in ICU (31.1%) and 43 (36.1%) died in hospital. Factors associated with hospital mortality by univariable and multivariable analyses are presented in Table 2.

Survivors were younger (63.5 [41.5–74] vs. 70 [50.5–76.5] years old), had fewer comorbidities, were less likely to be immunocompromised (30 out of 76 (39.5%) vs. 31 out of 43 (72.1%), *p* = 0.001), and a fewer of them had VZV disease occurring in ICU (3 out of 76 (3.9%) vs. 8 out of 43 (18.6%), *p* = 0.02).

By univariable analysis, other factors associated with hospital mortality were alcohol abuse, higher SOFA and SAPS II scores, higher leucocyte count on admission but lower leucocyte count in cerebrospinal fluid (CSF), use of norepinephrine, invasive mechanical ventilation, RRT, and occurrence of multiple ICU-acquired infections. Interestingly, the type of VZV-related organ injury was not associated with hospital mortality.

Five clinically relevant characteristics associated with hospital mortality in univariable analysis were included in the multivariable model (one explaining variable per eight events). Multivariable logistic regression analysis identified VZV disease occurring in ICU (OR 4.5 [1.02–25.36], *p* = 0.05), immunocompromised status (OR 3.43 [1.4–8.99], *p* = 0.01) and alcohol abuse (OR 4.48 [1.36–16.27], *p* = 0.02) as independent factors associated with hospital mortality (Table 2).

### Unsupervised clustering analysis

**Fig. 1:**
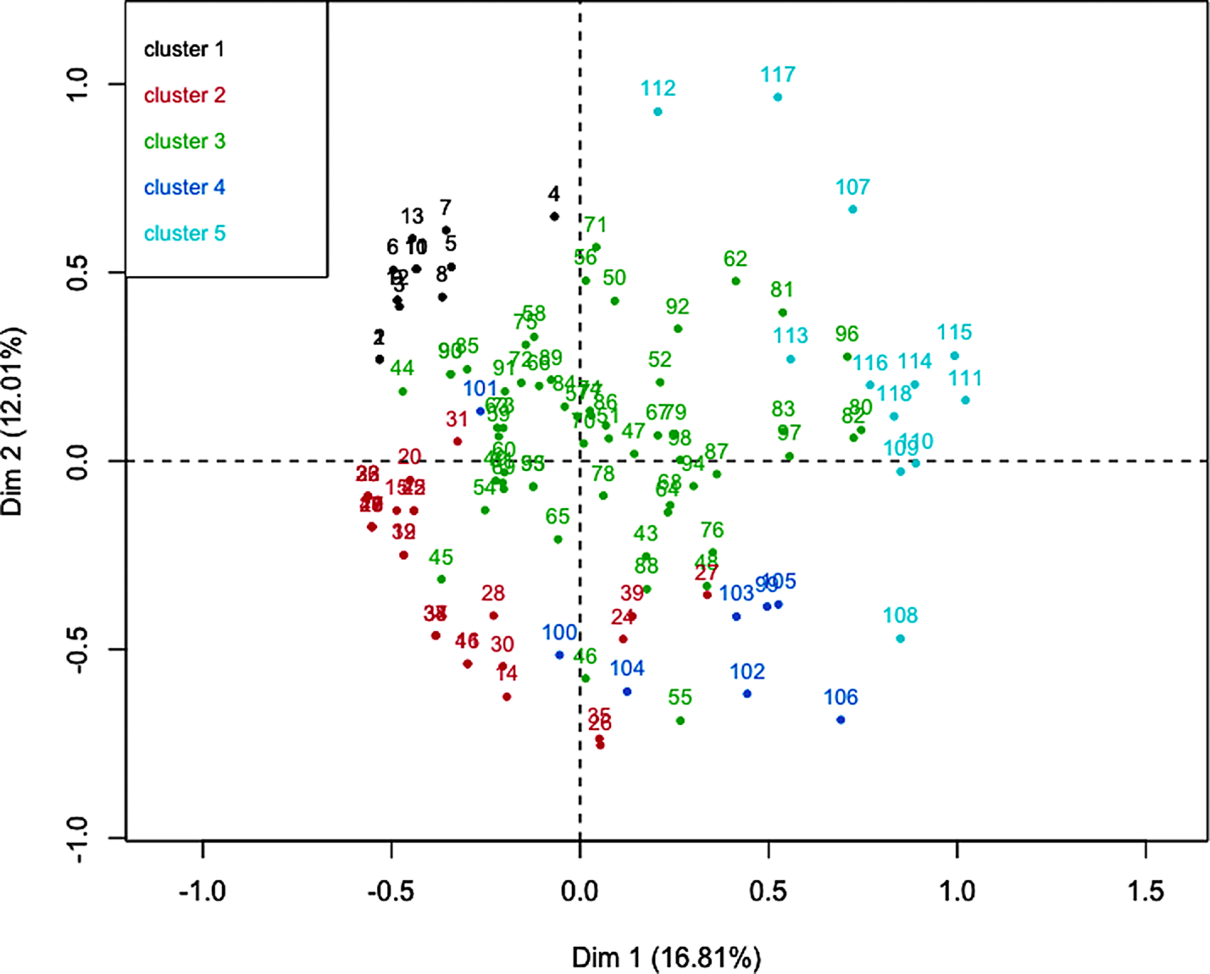
Factorial plan of hierarchical classification on principal components These first two dimensions summarize 20.8% of the data variability. The five clusters are represented with different colours. Black dots represent patients with mild encephalitis, red dots are for VZV-related pneumonia, green dots for severe encephalitis in immunocompromised patients, blue dots for VZV-disease occurring in ICU, and turquoise for severe encephalitis in solid organ transplant recipients

Clustering analysis led to the constitution of five clinically distinct phenotypes. The factorial plan, which represents a graphical distribution of patients and clusters is provided in Fig. 1 (with hierarchical tree in Additional file 2: Fig. 2). Detailed features of the five clusters are displayed in Table 3.

**Table 3:** Between-group differences based on clustering analysis

Characteristics	Cluster 1 (*n* = 13)	Cluster 2 (*n* = 29)	Cluster 3 (*n* = 56)	Cluster 4 (*n* = 8)	Cluster 5 (*n* = 12)	*P*
Age, *median [IQR]*	42 [27–76]	44 [36–54]	73 [62.5–79.25]	69.5 [46.75–75.5]	64.5 [56.75–68.25]	*< 0.001*
Female sex at birth, *n (%)*	5 (38.5)	7 (24.1)	27 (48.2)	1 (12.5)	5 (41.7)	*0.13*
SOFA score, *median [IQR]*	2 [1–4]	5 [2–8]	7 [3–9]	7 [2.25–9.75]	8.5 [5–10.25]	*0.006*
SAPS II score, *median [IQR]*	20 [12–34]	26 [18–37]	53.5 [35–66.75]	45 [37–56.5]	36 [32.5–51]	*< 0.001*
Hypertension, *n (%)*	3 (23.1)	4 (13.8)	33 (58.9)	4 (50)	7 (58.3)	*< 0.001*
Alcohol abuse, *n (%)*	1 (7.7)	3 (10.3)	7 (12.5)	2 (25)	5 (41.7)	*0.09*
Liver cirrhosis, *n (%)*	0 (0)	1 (3.4)	1 (1.8)	0 (0)	8 (66.7)	*< 0.001*
Chronic kidney disease, *n (%)*	0 (0)	1 (3.4)	9 (16.1)	2 (25)	7 (58.3)	*< 0.001*
Type 2 diabetes, *n (%)*	0 (0)	0 (0)	13 (23.2)	1 (12.5)	4 (33.3)	*0.003*
Immunocompromised^1^, *n (%)*	3 (23.1)	6 (20.7)	35 (62.5)	4 (50)	12 (100)	*< 0.001*
Solid tumour, *n (%)*	0 (0)	3 (10.3)	5 (8.9)	2 (25)	2 (16.7)	*0.32*
Haematologic malignancy, *n (%)*	2 (15.4)	1 (3.4)	17 (30.4)	0 (0)	2 (16.7)	*0.02*
Autoimmune disease, *n (%)*	1 (7.7)	1 (3.4)	14 (25)	1 (12.5)	1 (8.3)	*0.08*
Solid organ transplant, *n (%)*	0 (0)	0 (0)	1 (1.8)	0 (0)	12 (100)	*< 0.001*
Corticosteroids, *n (%)*	1 (7.7)	1 (3.4)	14 (25)	2 (25)	9 (75)	*< 0.001*
Immunosuppressive drugs, *n (%)*	0 (0)	1 (3.4)	11 (19.6)	0 (0)	12 (100)	*< 0.001*
Time between hospital presentation and ICU admission^2^ (days)	0 [0–0]	0 [0–2]	2.5 [1–6]	-	22 [3–23]	*< 0.001*
Time between hospital presentation and first symptoms^2^ (days)	-2 [-4 – -1]	-3 [-4.5 – -1.5]	-1 [-3–0]	-	16 [3–21]	*0.02*
Other Herpesviridae reactivation, *n (%)*	0 (0)	1 (3.4)	6 (10.7)	3 (37.5)	1 (8.3)	*0.07*
VZV disease occurring in ICU, *n (%)*	0 (0)	0 (0)	0 (0)	8 (100)	3 (25)	*< 0.001*
***VZV-related organ injury***						
Vesicular skin rash, *n (%)*	10 (76.9)	29 (100)	36 (64.3)	6 (75)	7 (58.3)	*0.005*
Encephalitis, *n (%)*	10 (76.9)	2 (6.9)	42 (75)	2 (25)	10 (83.3)	*< 0.001*
Pancreatitis, *n (%)*	0 (0)	0 (0)	5 (8.9)	0 (0)	0 (0)	*0.41*
Hepatitis, *n (%)*	3 (23.1)	1 (3.4)	5 (8.9)	2 (25)	0 (0)	*0.1*
Pneumonia, *n (%)*	0 (0)	29 (100)	16 (28.6)	4 (50)	3 (25)	*< 0.001*
ARDS, *n (%)*	0 (0)	16 (55.2)	5 (8.9)	3 (37.5)	1 (8.3)	*< 0.001*
***In-ICU management***						
Norepinephrine, *n (%)*	1 (7.7)	13 (44.8)	28 (50)	6 (75)	5 (41.7)	*0.02*
Invasive mechanical ventilation, *n (%)*	0 (0)	13 (44.8)	43 (76.8)	7 (87.5)	9 (75)	*< 0.001*
Neuromuscular blockers, *n (%)*	0 (0)	12 (41.4)	3 (5.4)	2 (25)	0 (0)	*< 0.001*
Renal replacement therapy, *n (%)*	0 (0)	7 (24.1)	14 (25)	5 (62.5)	5 (41.7)	*0.01*
Antiviral drugs, *n (%)*	13 (100)	29 (100)	56 (100)	7 (87.5)	12 (100)	*0.07*
Time between hospital presentation and first antiviral infusion^2^ (days)	0 [0–0]	0 [0–1]	2 [1–5]	-	20 [3–26]	*< 0.001*
Hospital-acquired infection, *n (%)*	0 (0)	11 (37.9)	21 (37.5)	4 (50)	8 (66.7)	*0.005*
Withholding or withdrawing of life-sustaining therapies, *n (%)*	0 (0)	0 (0)	9 (16.1)	2 (25)	3 (25)	*0.64*
***Prognosis***						
ICU mortality, *n (%)*	0 (0)	3 (10.3)	20 (35.7)	5 (62.5)	8 (66.7)	*< 0.001*
Hospital mortality, *n (%)*	0 (0)	3 (10.3)	25 (44.6)	6 (75)	8 (66.7)	*< 0.001*

^1^Defined as ongoing solid tumour or cured less than 5 years prior, hematologic malignancy, autoimmune disease, solid organ transplant, primary immune deficit, HIV infection, corticosteroids, or immunosuppressive drugs

^2^VZV disease occurring in ICU excluded

The first cluster (*n* = 13) was composed of young patients (median age 42 [27–76]) without comorbidities. ICU admission was mainly due to VZV encephalitis and occurred on the day of hospital presentation. It was the least severe phenotype with a median SOFA score of 2 (IQR 1–4) and no hospital mortality.

The second cluster (*n* = 29) included young patients (median age 4 [36–54] 4 years old) quickly admitted for VZV-related pneumonia. All patients presented with typical diffuse vesicular skin rash. Sixteen out of 29 (55.2%) met the Berlin definition for ARDS. Invasive mechanical ventilation and neuromuscular blockers were used in 44.8% and 41.4%, respectively. Three patients ultimately needed venovenous extracorporeal membrane oxygenation. Hospital mortality rate was low at 10.3%.

Cluster 3 (*n* = 56) was characterized by severe encephalitis requiring invasive mechanical ventilation (76.8%). Thirty-five patients in this cluster (62.5%) were immunocompromised, mainly due to haematologic malignancies or autoimmune diseases. Hospital mortality rate was elevated (44.6%).

Cluster 4 (*n* = 8) was the more severe phenotype with a 75% hospital mortality rate. All members of this cluster had onset of VZV disease during their ICU stay. There was no specific organ injury, and half of them were considered immunocompromised at admission. Other *Herpesviridae* were isolated from laboratory samples for 37.5% of them and all patients but one were placed under mechanical ventilation.

Cluster 5 (*n* = 12) consisted of very severe encephalitis (as assessed by higher severity scores on admission (SAPS II and SOFA) comparing to the other phenotypes, and a hospital mortality rate of 66.7%) occurring in deeply immunocompromised hosts. All patients in this cluster were solid organ transplant recipients and were treated with immunosuppressive drugs. A typical vesicular skin rash was present in only 58.3% of patients and ICU admission occurred after prolonged hospitalization (22 [3–23] days). Most patients presented first symptoms while they were already hospitalized in conventional wards. Eight out of 12 patients (66.7%) experienced at least one episode of ICU-acquired infection.

A sensitivity analysis was performed after exclusion of diagnosis relying on clinical examination only (23 patients without molecular detection of VZV, despite all presenting with typical diffuse vesicular skin rash making the diagnosis rather undoubtful). In these conditions, the sensitivity analysis led, however, to the same results (Additional file 2: Table 3 and Additional file 2: Fig. 3).

## Discussion

The VAZOREA study is the largest cohort focusing on overall events related to varicella-zoster virus and requiring ICU admission, with the aim of providing data on clinical and biological presentation, in-ICU management, and hospital prognosis. Our clustering analysis provided five objectively diverse phenotypes of VZV disease, mainly defined by their host profile and with highly different hospital mortality. By multivariable analysis, independent factors associated with hospital mortality were VZV disease occurring in ICU, an immunocompromised status and alcohol abuse.

As expected, organ involvements were dominated by encephalitis and pneumonia which are two well-recognized severe complications of VZV infection [1, 6, 39]. Our unsupervised clustering analysis provided five phenotypes with very different organ injury and prognosis. Patients with VZV-related pneumonia and no encephalitis (cluster 2) were young. All presented with typical diffuse vesicular skin rash, and ICU-mortality was low. Overall, characteristics of this cluster were similar to those of another retrospective cohort study on VZV-related pneumonia, based on 102 patients from 29 French ICUs [22].

As acute viral encephalitis often leads to ICU admission for monitoring and treatment [40], an epidemiological study in the ICU setting may overestimate the real incidence of neurological complications but may provide valuable information on at-risk patients. Clustering analysis revealed three different phenotypes of encephalitis. The first one (cluster 1) occurred in younger and less immunocompromised patients, without any comorbidities. No patient but one needed organ support in cluster 1, while in cluster 3 and 5, nearly half of patients required norepinephrine (50% and 41.7% respectively), and three quarters were intubated. Consequently, ICU and hospital mortality rates were far higher in clusters 3 and 5 than in cluster 1. As previously published in a cohort of 55 patients with VZV encephalitis in ICU [27], the typical vesicular skin rash can be missing, especially in the most severe form of disease. Such an atypical presentation may cause a time delay in disease recognition and initiation of antiviral therapy. In our cohort, skin rash was absent in nearly half of the patients from cluster 5. These patients also had a longer time to ICU admission and first antiviral infusion. In a recent international cohort study of all-causes encephalitis in ICU (EURECA), a delay in acyclovir initiation was independently associated with worse outcome [41]. This raises the question of whether high-risk patients, and in particular solid organ transplant recipients, might benefit from prophylactic antiviral treatment, which is not currently recommended (except for allogeneic stem cell transplant recipients [42]). Similarly, although literature data on the recombinant herpes zoster vaccine are scarce in solid organ transplant recipients, the safety and immunogenicity of this vaccine have now been demonstrated [43, 44], so this could be a game-changer in the future.

Overall, 51.3% of patients were immunocompromised. This result is in line with a Danish cohort of hospitalized patients with VZV encephalitis [25] and a large cohort of patients with herpes simplex virus (HSV) encephalitis admitted in ICU [45]. In the present study, CSF leucocyte count was lower in non-survivors in univariable analysis, and might be a surrogate marker for immunosuppression, which was identified as a main factor independently associated with poor outcome. This finding on immunosuppression is consistent with the EURECA study [41], but was not found in another retrospective study focusing on VZV encephalitis [27], possibly due to selection bias. Even when narrowing on encephalitis our immunosuppression rate is 59%, which is far less than the 78% of immunocompromised patients reported by *Mirouse & al.* [27], despite similar definitions. In this study, 18 centres were involved but some were highly specialized in immunocompromised patients and might have led to an overrepresentation of immunosuppression among VZV encephalitis. Our cohort study involved 26 centres, academic as well as non-academic, with no centre highly specialized in the management of immunocompromised patients. Thus, our study design and our immunosuppression rate similar to that of a nationwide cohort study [25] may demonstrate that our study is more representative of a general ICU population. The unsupervised clustering analysis also supports the fact that hospital mortality is mainly driven by immunosuppression status rather than by any organ involvement. Clusters 3 to 5, the most associated with poor prognosis, are mainly characterized by profound immunosuppression responsible for severe VZV disease. Cluster 5 was characterized by deeply immunocompromised patients treated with systemic corticosteroids or immunosuppressive drugs. These therapies impair T-cell mediated immunity, which has proven critical against VZV [1, 3]. Noteworthy, occurrence of VZV disease during ICU stay was associated with high mortality and may be related – at least in part – to post-aggressive immune dysfunction, an emerging concept in ICU patients [46–49].

During VZV encephalitis, use of systemic corticosteroids is still a matter of debate [38, 50]. Adjunctive corticosteroids may be an effective therapy against VZV-associated vasculitis [3]. However, in our study as well as in two others use of systemic corticosteroids was not associated with improved outcome [25, 27]. An ongoing randomized controlled trial evaluating adjunctive dexamethasone in HSV encephalitis will provide more information on the potential positive or detrimental effects of corticosteroids in viral encephalitis (NCT03084783).

Interestingly, our study found alcohol consumption, even moderate, as an independent factor associated with mortality. In the past few years, several prospective cohort studies tended to demonstrate that moderate alcohol consumption was associated with poorer outcome, in ICU but also up to one year after ICU admission [51, 52].

Our study has several strengths. First, to our knowledge, this is the largest cohort study on critically ill patients with VZV-associated disease. The number of centres involved in the study and their diversity in size and academics provide data on real-life practices and add relevant data on the understanding of VZV-associated disease. Second, in contrast with the existing literature [22, 25, 27] this study was not restricted to any specific organ involvement and allowed us to depict the global picture of VZV disease in ICU. Thanks to wide eligibility criteria we were able to carry out a clustering analysis without any prior cognitive bias, thus allowing us to identify the objectively diverse phenotypes of VZV disease in ICU patients, which are more accurately defined by their host profile than their specific organ involvement. This may help clinicians recognize high-risk patients and assess prognosis.

This study also has several limitations. As in all observational studies, heterogeneity of patient care between centres could have affected the results on prognosis. Despite their growing interest in sepsis and septic shock, long-term prognosis and health-related quality of life were not assessed in the present study. Considering literature data on sepsis [53–57], survivors of a severe VZV-related event may suffer from long-term impairment of their respiratory and cognitive functions, thus resulting in disability. In their study on VZV encephalitis, *Mirouse et al.* found that only 36% of patients had favourable neurologic outcome (modified Rankin scale 0–2) one year after ICU admission. In another French retrospective cohort on neurologic VZV infection, patients with encephalitis had even a poorer prognosis with 82% of unfavourable outcome (defined as death or any persistent symptom or sequelae) [58]. All cases were reviewed by investigators, but definitions of specific organ injury were mainly based on medical charts and clinical judgement, in a “real-life” setting. This retrospective design cannot rule out misclassification. However, all cases included without VZV isolation in laboratory samples (virologic tests not performed) were characterized by diffuse vesicular skin rash typical of VZV disease. Moreover, sensitivity analysis performed after exclusion of patients without virologic evidence of VZV led to the same results. There are currently no consensus criteria for definite VZV-related pneumonia and isolation of VZV DNA in respiratory samples does not mean VZV disease [48, 59]. In a recently published retrospective monocentric study in ICU, *Guiraud et al.* found that VZV isolation in bronchoalveolar lavage was not associated with respiratory failure but with shingles occurrence [59]. As systematically performed biopsies to demonstrate cytopathic effect are not feasible, studies aiming to find criteria for VZV-related pneumonia based on a combination of clinical and laboratory criteria would be of great interest.

In summary, we here report the largest study on critically ill patients with VZV-related events. Our unsupervised clustering analysis revealed five distinct groups of patients, with highly different hospital mortality rates. Together with the independent predictors of poor outcome identified, these results are critical to help the clinicians recognize high-risk patients and assess prognosis.

### Electronic supplementary material

Below is the link to the electronic supplementary material.

Supplementary Material 1

Supplementary Material 2

## Data Availability

All data and code are available after request to JM and DDC.

## Abbreviations

ARDS: acute respiratory distress syndrome
CSF: cerebrospinal fluid
DNA: deoxyribonucleic acid
HCPC: hierarchical clustering on principal components
HIV: human immunodeficiency virus
ICU: intensive care unit
IQR: interquartile range
MCA: multiple component analysis
RRT: renal replacement therapy
SAPS: simplified acute physiology score
SOFA: sequential organ failure assessment
STROBE: Strengthening the Reporting of Observational Studies in Epidemiology
VZV: Varicella-zoster virus
