# Supplementary material for: Clinical description and outcome of overall varicella-zoster virus-related organ dysfunctions admitted in intensive care units: the VAZOREA cohort study
Source: Ann Intensive Care. 2024 Mar 29;14:44. doi: 10.1186/s13613-024-01270-w (PMC10978565; doi:10.1186/s13613-024-01270-w)
Supplement: Supplementary file 2 — Supplementary Material 2 [file 13613_2024_1270_MOESM2_ESM.docx]

**Supplementary Table 1 VAZOREA participating intensive care units**

| **Centre** | **Patients, *n (%)*** |
| --- | --- |
| Bordeaux | 16 (13.4) |
| La Roche sur Yon | 8 (6.7) |
| Caen | 7 (5.9) |
| Lille | 7 (5.9) |
| Rennes | 7 (5.9) |
| Clermont-Ferrand | 6 (5.0) |
| Lyon Est | 6 (5.0) |
| Nice | 6 (5.0) |
| Strasbourg | 6 (5.0) |
| Cochin | 5 (4.2) |
| Lyon Sud | 5 (4.2) |
| Orléans | 5 (4.2) |
| Argenteuil | 4 (3.4) |
| Dijon | 4 (3.4) |
| Henri Mondor | 4 (3.4) |
| Vannes | 4 (3.4) |
| Tourcoing | 3 (2.5) |
| Saint Antoine | 3 (2.5) |
| Angoulême | 2 (1.7) |
| Le Mans | 2 (1.7) |
| Nancy | 2 (1.7) |
| Poitiers | 2 (1.7) |
| Saint-Nazaire | 2 (1.7) |
| Cherbourg | 1 (0.8) |
| La Rochelle | 1 (0.8) |
| Saint-Denis (La Réunion) | 1 (0.8) |

**Supplementary Table 2 Detailed initial and in-ICU characteristics of the 119 patients with severe VZV events**

| **Characteristics** |  |
| --- | --- |
| Age, *median [IQR]* | 66 [45–75] |
| Female sex at birth, *n (%)* | 46 (38.7) |
| BMI (kg/m^2^), *median [IQR]* | 25.1 [22.35–29.1] |
| SOFA score | 6 [2–9] |
| SAPS II score | 40 [26–60.25] |
| VZV disease occurring in ICU, *n (%)* | 11 (9.2) |
| ***Comorbidities*** | |
| Hypertension, *n (%)* | 51 (42.9) |
| Chronic heart failure, *n (%)* | 14 (11.8) |
| Alcohol abuse, *n (%)* | 18 (15.1) |
| Liver cirrhosis, *n (%)* | 10 (8.4) |
| Chronic kidney disease, *n (%)* | 19 (16) |
| Smokers, *n (%)* | 48 (40.3) |
| COPD, *n (%)* | 13 (10.9) |
| Asthma, *n (%)* | 8 (6.7) |
| Inhaled corticosteroids, *n (%)* | 10 (8.4) |
| Chronic respiratory failure, *n (%)* | 7 (5.9) |
| Type 2 diabetes mellitus, *n (%)* | 18 (15.1) |
| Immunocompromised^1^, *n (%)* | 61 (51.3) |
| Solid tumour, *n (%)* | 12 (10.1) |
| Prostate, *n (%)* | 3 (25) |
| Lung, *n (%)* | 2 (16.7) |
| Breast, *n (%)* | 2 (16.7) |
| Colon, *n (%)* | 1 (8.3) |
| Kidney, *n (%)* | 1 (8.3) |
| Liver, *n (%)* | 1 (8.3) |
| Bladder, *n (%)* | 1 (8.3) |
| Testis, *n (%)* | 1 (8.3) |
| On chemotherapy, *n (%)* | 6 (5) |
| Radiotherapy, *n (%)* | 5 (4.2) |
| Immunotherapy, *n (%)* | 10 (8.4) |
| Haematologic malignancy, *n (%)* | 23 (19.3) |
| Non-Hodgkin lymphoma, *n (%)* | 8 (34.8) |
| Chronic lymphocytic leukaemia, *n (%)* | 5 (21.7) |
| Waldenström, *n (%)* | 4 (17.4) |
| Acute myeloid leukaemia, *n (%)* | 1 (4.3) |
| Myelodysplastic syndrome, *n (%)* | 1 (4.3) |
| Myeloproliferative syndrome, *n (%)* | 1 (4.3) |
| Multiple myeloma, *n (%)* | 1 (4.3) |
| T cell lymphoma, *n (%)* | 1 (4.3) |
| Acute lymphoid leukemia | 1 (4.3) |
| Allogenic stem cell transplant | 1 (0.8) |
| Autoimmune disease, *n (%)* | 18 (15.1) |
| Small vessel vasculitis, *n (%)* | 7 (41.2) |
| Rheumatoid arthritis, *n (%)* | 3 (17.6) |
| Systemic lupus erythematosus, *n (%)* | 2 (11.8) |
| Dermatomyositis, *n (%)* | 1 (5.9) |
| Inflammatory bowel disease, *n (%)* | 1 (5.9) |
| Ankylosing spondylitis, *n (%)* | 1 (5.9) |
| Acquired haemophilia, *n (%)* | 1 (5.9) |
| Other inflammatory rheumatism, *n (%)* | 1 (5.9) |
| Solid organ transplant, *n (%)* | 13 (10.9) |
| Liver, *n (%)* | 8 (61.5) |
| Kidney, *n (%)* | 6 (46.2) |
| Heart, *n (%)* | 1 (7.7) |
| Primitive immune deficiency, *n (%)* | 1 (0.8) |
| Corticosteroids, *n (%)* | 27 (22.7) |
| Dosing regimen (mg/day) | 10 [5–20] |
| Immunosuppressive drugs, *n (%)* | 24 (20.2) |
| Number of immunosuppressive drugs, *n (%)* |  |
| 1 | 15 (62.5) |
| 2 | 8 (33.3) |
| 3 | 1 (4.2) |
| Pregnancy, *n (%)* | 4 (3.4) |
| HIV, *n (%)* | 4 (3.4) |
| ***On admission*** | |
| Time between hospital presentation and ICU admission (days) | 2 [0–5] |
| Main reason for ICU admission, *n (%)* |  |
| Neurologic failure | 51 (42.9) |
| Respiratory failure | 45 (37.8) |
| Multiple organ failure | 10 (8.4) |
| Sepsis alone | 7 (5.9) |
| Circulatory shock | 4 (3.4) |
| Liver failure | 1 (0.8) |
| Other | 1 (0.8) |
| VZV-related organ injury, *n (%)* |  |
| Vesicular skin rash | 89 (74.8) |
| Encephalitis | 66 (55.5) |
| Laboratory confirmed | 62/66 (93.9) |
| Positive PCR on CSF | 60/66 (90.1) |
| Leucocyte count (/mm^3^)^3^ | 76 [15.5–223] |
| CSF protein count (g/L)^3^ | 0.96 [0.62–1.89] |
| CSF glucose count (ratio of glycemia)^4^ | 0.55 [0.42–0.67] |
| Blood viral load | 1/66 (1.5) |
| Positive PCR on skin biopsy | 1/66 (1.5) |
| Pneumonia | 53 (44.5) |
| Laboratory confirmed | 36/53 (67.9) |
| Positive PCR on CSF | 6/53 (11.3) |
| Both blood viral load and positive PCR on respiratory sample | 13/53 (24.5) |
| Blood viral load only | 4/53 (7.5) |
| Positive PCR on skin biopsy | 7/53 (13.2) |
| Immunofluorescence on skin biopsy | 1/53 (1.9) |
| Positive PCR on respiratory sample only | 4/53 (7.5) |
| Other | 1/53 (1.9) |
| ARDS | 26 (21.8) |
| Mild | 4 (15.4) |
| Moderate | 6 (23.1) |
| Severe | 16 (61.5) |
| Hepatitis | 11 (9.2) |
| Laboratory confirmed | 9/11 (81.8) |
| Positive PCR on CSF | 2/11 (18.2) |
| Blood viral load | 6/11 (54.5) |
| Positive PCR on skin biopsy | 1/11 (9.1) |
| Isolated | 3/11 (27.3) |
| Severe hepatitis (prothrombin time < 50%) | 6/11 (54.5) |
| Pancreatitis | 5 (4.2) |
| Laboratory confirmed | 4/5 (80) |
| Blood viral load | 3/5 (60) |
| Positive PCR on CSF | 1/5 (20) |
| Sepsis | 105 (88.2) |
| Septic shock | 18 (15.1) |
| Contact with chickenpox < 21 days, *n (%)* | 20 (16.8) |
| ***Laboratory values on ICU admission*** | |
| \| Lactate (mmol/L) \| \| --- \| | 1.5 [1–2.5] |
| Leucocyte count (/mm^3^) | 9415 [6825–13100] |
| Neutrophil count (/mm^3^) | 6745 [3742.5–9665] |
| Lymphocyte count (/mm^3^) | 845 [378.25–1670] |
| Platelets (G/L) | 156 [90–240.25] |
| Creatinine (µmol/L) | 87 [62–150] |
| Bilirubin (µmol/L) | 11 [7–19] |
| Prothrombin time (%) | 80 [64–92.25] |
| ***Other Herpesviridae viraemia, n (%)*** | 11 (9.2) |
| CMV | 3 (27.3) |
| EBV | 5 (45.5) |
| HHV6 | 1 (9.1) |
| HSV | 2 (18.2) |
| Initiation of antiviral treatment for Herpesviridae viraemia, n (%) | 2 (1.7) |
| ***In ICU management*** | |
| Use of norepinephrine, *n (%)* | 54 (45.4) |
| Time between ICU admission and norepinehrine infusion (days) | 0 [0–1] |
| Norepinephrine maximum dose (µg/kg/min) | 0.42 [0.22–0.95] |
| Duration of circulatory support (days) | 3 [1–5] |
| Use of dobutamine, *n (%)* | 3 (2.5) |
| Time between ICU admission and dobutamine infusion (days) | 1 [0.5–4.5] |
| Maximum dose of dobutamine (µg/kg/min) | 5 [5–5] |
| Duration of dobutamine infusion (days) | 2 [1.5–2.5] |
| Use of any respiratory support, *n (%)* | 99 (83.9) |
| Standard oxygen alone, *n (%)* | 18 (18.2) |
| Maximum oxygen flow rate (L/min) | 3 [2–5] |
| Duration of standard oxygen (days) | 3.5 [2–5.75] |
| High-flow nasal oxygen (HFNO), *n (%)* | 5 (5.1) |
| Time between ICU admission and HFNO initiation (days) | 1 [0–3] |
| Maximum FiO_2_ (%) | 95 [40–100] |
| Duration of HFNO (days) | 4 [2–4] |
| Non-invasive ventilation (NIV), *n (%)* | 4 (4) |
| Time between ICU admission and NIV initiation (days) | 0 [0–0] |
| Maximum FiO_2_ (%) | 80 [68.75–85] |
| Duration of NIV (days) | 7.5 [4.25–10.25] |
| Invasive mechanical ventilation, *n (%)* | 72 (72.7) |
| Time between ICU admission and intubation (days) | 0 [0–1] |
| Maximum FiO_2_ (%) | 70 [50–100] |
| Duration of invasive mechanical ventilation (days) | 10 [4.75–20] |
| Use of neuromuscular blockers, *n (%)* | 17 (17.2) |
| Time between ICU admission and use of neuromuscular blockers (days) | 1 [0–1] |
| Duration of neuromuscular blockers infusion (days) | 3 [2–6] |
| Prone positioning, *n (%)* | 7 (7.1) |
| Time between ICU admission and prone position (days) | 1 [0.5–4.5] |
| Duration of prone positioning (days) | 4 [1–4.5] |
| Venovenous ECMO, *n (%)* | 6 (6.1) |
| Time between ICU admission and use of VV-ECMO (days) | 3.5 [2.25–4] |
| Duration of VV-ECMO (days) | 6.5 [4–15] |
| Renal-replacement therapy (RRT), *n (%)* | 31 (26.3) |
| Time between ICU admission and RRT (days) | 0 [0–1] |
| Duration of RRT (days) | 13 [4–31] |
| Use of antiviral drugs, *n (%)* | 117 (98.3) |
| Acyclovir, *n (%)* | 113 (96.6) |
| Valacyclovir, *n (%)* | 3 (2.6) |
| Ganciclovir, n (%) | 1 (0.9) |
| Dose of acyclovir (mg/kg/day) | 30 [30–45] |
| Time between hospital admission and use of antiviral drugs (days)^2^ | 1 [0–5] |
| Time between ICU admission and use of antiviral drugs (days)^2^ | 0 [0–1] |
| Time between first symptoms and use of antiviral drugs (days) | 3 [1–6] |
| Duration of antiviral treatment (days) | 11 [7–19] |
| Correct antiviral dosing regimen, *n (%)* | 76 (68.5) |
| Use of systemic corticoids for VZV infection, *n (%)* | 10 (8.7) |
| VZV-related pneumonia | 3 (30) |
| Brain vasculitis | 7 (70) |
| ICU-acquired infections, *n (%)* |  |
| At least one episode | 44 (37) |
| At least two episodes | 12 (10.2) |
| At least three episodes | 7 (5.9) |
| Time between ICU admission and first nosocomial infection (days) | 4 [2–9] |
| Time between ICU admission and second nosocomial infection (days) | 14.5 [9.5–27] |
| Time between ICU admission and third nosocomial infection (days) | 33 [18–67] |
| Duration of anti-infectious drugs for the first episode (days) | 7 [5–11] |
| Duration of anti-infectious drugs for the second episode (days) | 7 [7–7.5] |
| Duration of anti-infectious drugs for the third episode (days) | 12 [7.5–16.5] |
| ***Prognosis*** | |
| ICU mortality, *n (%)* | 37 (31.1) |
| Withholding/withdrawing of life sustaining therapies, *n (%)* | 15 (40.5) |
| Time between ICU admission and death (days) | 16 [5–31] |
| Hospital mortality, *n (%)* | 43 (36.1) |
| Time between hospital admission and death (days) | 23 [12–50] |
| Cause of in-hospital deaths, *n (%)* |  |
| Refractory shock/refractory multiple organ failure | 24 (55.8) |
| Persistent comatose state | 12 (27.9) |
| Brain death | 2 (4.7) |
| Refractory hypoxemia | 4 (9.3) |
| Acute kidney injury, n (%) | 63 (58.3) |

^1^Defined as ongoing solid tumour or cured less than 5 years prior, hematologic malignancy, autoimmune disease, solid organ transplant, primary immune deficit, HIV infection, corticosteroids, or immunosuppressive drugs
^2^VZV disease occurring in ICU excluded
^3^Four missing values
^4^31 missing values

**Supplementary Table 3 Sensitivity analysis of unsupervised clustering**

| **Characteristics** | **Cluster 1 (n=16)** | **Cluster 2 (n=20)** | **Cluster 3 (n=37)** | **Cluster 4 (n=6)** | **Cluster 5 (n=16)** | ***p*** |
| --- | --- | --- | --- | --- | --- | --- |
| Age, *median [IQR]* | 67 [29.25–76.25] | 47.5 [42.75–64.5] | 74 [72–82] | 56.5 [46.25–71.25] | 63 [57.75–68.5] | *<0.001* |
| Female sex at birth, *n (%)* | 8 (50) | 5 (25) | 20 (54.1) | 1 (16.7) | 5 (31.2) | *0.13* |
| SOFA score, *median [IQR]* | 3.5 [1.75–5] | 5 [2–8.75] | 7 [3–8] | 6 [0.75–11.25] | 10 [8.75–12] | *<0.001* |
| SAPS II score, *median [IQR]* | 29 [18.25–39] | 28 [24.25–47] | 55 [40.5–65] | 40 [31–47.5] | 60 [38–66] | *<0.001* |
| Hypertension, *n (%)* | 5 (31.2) | 2 (10) | 25 (67.6) | 2 (33.3) | 11 (68.8) | *<0.001* |
| Alcohol abuse, *n (%)* | 1 (6.2) | 2 (10) | 2 (5.4) | 2 (33.3) | 7 (43.8) | *0.004* |
| Liver cirrhosis, *n (%)* | 1 (6.2) | 0 (0) | 1 (2.7) | 0 (0) | 6 (37.5) | *0.001* |
| Chronic kidney disease, *n (%)* | 3 (18.8) | 1 (5) | 5 (13.5) | 2 (33.3) | 7 (43.8) | *0.03* |
| Type 2 diabetes, *n (%)* | 4 (25) | 0 (0) | 6 (16.2) | 0 (0) | 6 (37.5) | *0.02* |
| Immunocompromised^1^, *n (%)* | 7 (43.8) | 7 (35) | 20 (54.1) | 3 (50) | 15 (93.8) | *0.01* |
| Solid tumour, *n (%)* | 2 (12.5) | 0 (0) | 3 (8.1) | 1 (16.7) | 1 (6.2) | *0.4* |
| Haematologic malignancy, *n (%)* | 4 (25) | 2 (10) | 12 (32.4) | 0 (0) | 1 (6.2) | *0.01* |
| Autoimmune disease, *n (%)* | 1 (6.2) | 5 (25) | 2 (5.4) | 1 (16.7) | 9 (56.2) | *<0.001* |
| Solid organ transplant, *n (%)* | 3 (18.8) | 0 (0) | 1 (2.7) | 0 (0) | 8 (50) | *<0.001* |
| Corticosteroids, *n (%)* | 5 (31.2) | 3 (15) | 5 (13.5) | 2 (33.3) | 10 (62.5) | *0.004* |
| Immunosuppressive drugs, *n (%)* | 3 (18.8) | 4 (20) | 3 (8.1) | 0 (0) | 13 (81.2) | *<0.001* |
| Other Herpesviridae reactivation, *n (%)* | 0 (0) | 1 (5) | 5 (13.5) | 3 (50) | 0 (0) | *0.01* |
| VZV disease occurring in ICU, *n (%)* | 2 (12.5) | 0 (0) | 0 (0) | 6 (100) | 1 (6.2) | *<0.001* |
| ***VZV-related organ injury*** | | | | | | |
| Vesicular skin rash, *n (%)* | 11 (68.8) | 19 (95) | 22 (59.5) | 4 (66.7) | 9 (56.2) | *0.06* |
| Encephalitis, *n (%)* | 15 (93.8) | 1 (5) | 32 (86.5) | 2 (33.3) | 14 (87.5) | *<0.001* |
| Pancreatitis, *n (%)* | 1 (6.2) | 0 (0) | 3 (8.1) | 0 (0) | 0 (0) | *0.66* |
| Hepatitis, *n (%)* | 2 (12.5) | 2 (10) | 1 (2.7) | 2 (33.3) | 2 (12.5) | *0.1* |
| Pneumonia, *n (%)* | 0 (0) | 20 (100) | 9 (24.3) | 4 (66.7) | 3 (18.8) | *<0.001* |
| ARDS, *n (%)* | 0 (0) | 11 (55) | 3 (8.1) | 3 (50) | 1 (6.2) | *<0.001* |
| ***In-ICU management*** | | | | | | |
| Norepinephrine, *n (%)* | 2 (12.5) | 11 (55) | 17 (45.9) | 5 (83.3) | 9 (56.2) | *0.01* |
| Invasive mechanical ventilation, *n (%)* | 0 (0) | 12 (60) | 29 (78.4) | 6 (100) | 16 (100) | *0.01* |
| Neuromuscular blockers, *n (%)* | 0 (0) | 10 (50) | 2 (5.4) | 2 (33.3) | 0 (0) | *<0.001* |
| Renal replacement therapy, *n (%)* | 1 (6.2) | 6 (30) | 4 (10.8) | 5 (83.3) | 11 (68.8) | *<0.001* |
| Antiviral drugs, *n (%)* | 16 (100) | 20 (100) | 37 (100) | 5 (83.3) | 16 (100) | *0.06* |
| Hospital-acquired infection, *n (%)* | 1 (6.2) | 7 (35) | 14 (37.8) | 3 (50) | 11 (68.8) | *0.005* |
| Withholding or withdrawing of life-sustaining therapies, *n (%)* | 0 (0) | 1 (5) | 5 (13.5) | 1 (16.7) | 5 (31.2) | *0.94* |
| ***Prognosis*** | | | | | | |
| ICU mortality, *n (%)* | 1 (6.2) | 3 (15) | 11 (29.7) | 4 (66.7) | 11 (68.8) | *<0.001* |
| Hospital mortality, *n (%)* | 2 (12.5) | 3 (15) | 15 (40.5) | 5 (83.3) | 11 (68.8) | *<0.001* |

^1^Defined as ongoing solid tumour or cured less than 5 years prior, hematologic malignancy, autoimmune disease, solid organ transplant, primary immune deficit, HIV infection, corticosteroids, or immunosuppressive drugs


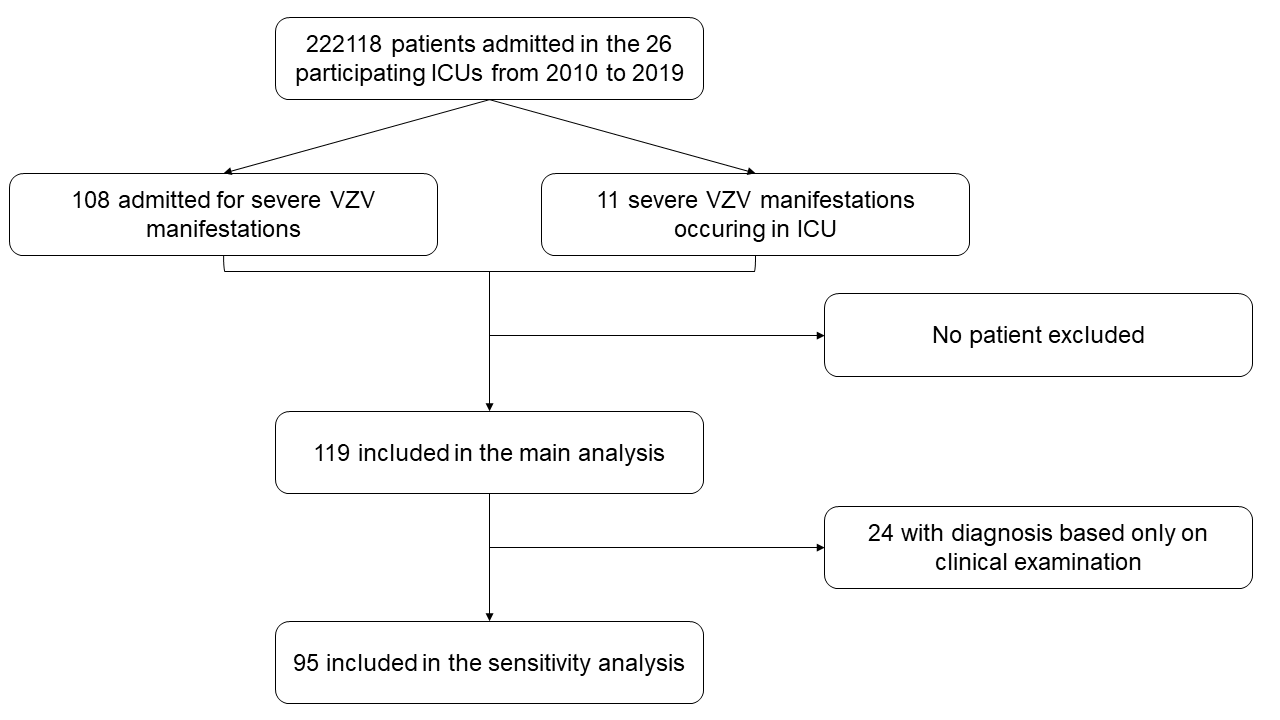


**Supplementary Fig.1** Cohort study flow chart

**
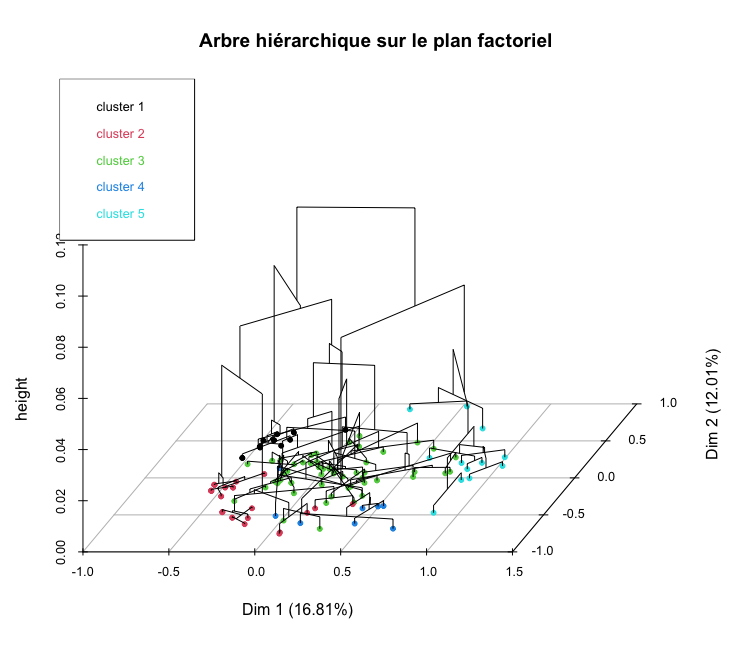
**

**Supplementary Fig.2** 3D surperimposition of hierarchical plan made with the first two dimensions and the hierarchical tree of classification

**
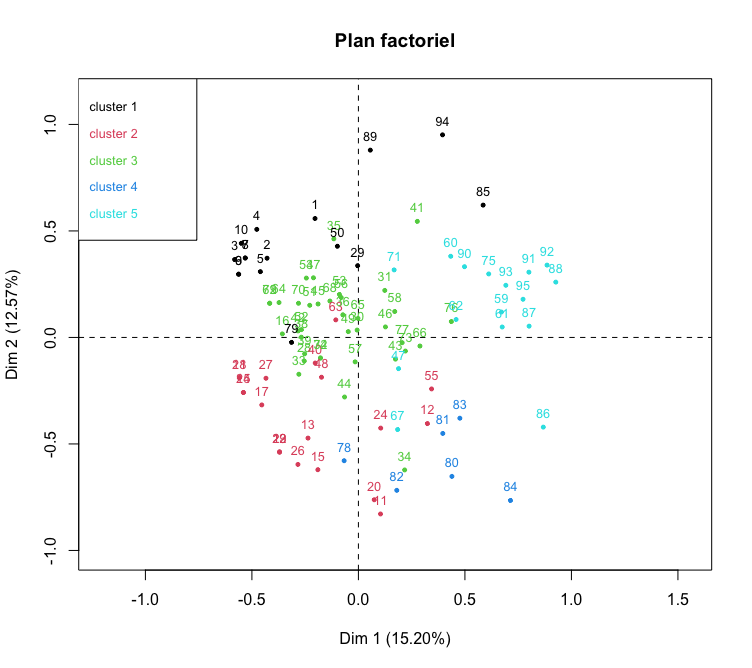
**

**Supplementary Fig.3** Factorial plan of sensitivity analysis
These first two dimensions summarize 27.8% of data variability


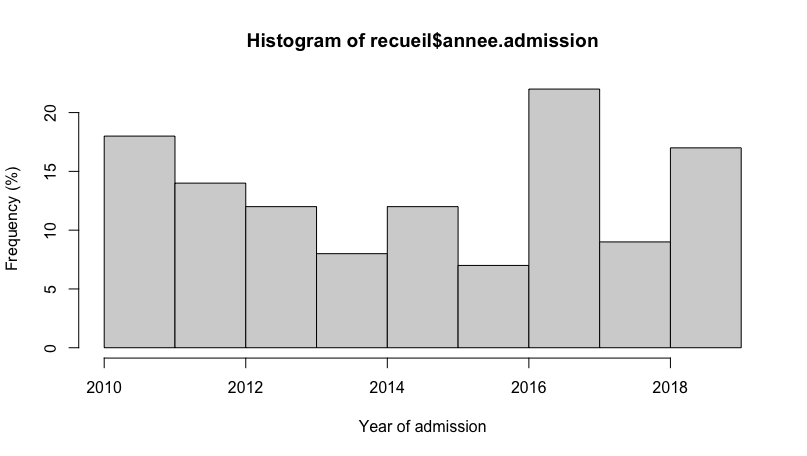


**Supplementary Fig.4** Histogram of frequency of cases across time
